# Comprehensive genomic profiling of a unique liposarcoma arising in a patient with Li–Fraumeni syndrome and the novel detection of c-myc amplification: a case report

**DOI:** 10.1186/s13000-022-01264-x

**Published:** 2022-12-13

**Authors:** Hirofumi Watanabe, Fumiyoshi Fujishima, Toru Motoi, Yayoi Aoyama, Tetsuya Niihori, Masanobu Takahashi, Sho Umegaki, Hisashi Oishi, Hiroshi Tada, Ryo Ichinohasama, Hironobu Sasano

**Affiliations:** 1 Department of Pathology, Tohoku University Hospital, 1-1 Seiryo-machi, Aoba-ku, Miyagi-ken, Sendai, Japan; 2 Department of Pathology, Tokyo Metropolitan Cancer and Infectious Diseases Center Komagome Hospital, Tokyo, Japan; 3 Department of Medical Genetics, Tohoku University School of Medicine, Sendai, Japan; 4 Department of Medical Oncology, Tohoku University Hospital, Sendai, Japan; 5 Department of Thoracic Surgery, Institute of Development, Aging and Cancer, Tohoku University, Sendai, Japan; 6 Department of Breast and Endocrine Surgical Oncology, Graduate School of Medicine, Tohoku University, Sendai, Japan; 7 Department of Hematopathology, Tohoku University Hospital, Sendai, Japan

**Keywords:** Li–Fraumeni syndrome, *TP53* mutation, *c-myc*, Myxoid pleomorphic liposarcoma, Cartilaginous differentiation

## Abstract

**Background:**

Germline *TP53* mutations have been frequently reported in patients with Li–Fraumeni syndrome (LFS), resulting in a predisposition to various malignancies. Mutations other than germline *TP53* mutations can also cause LFS-associated malignancies, but their details remain unclear. We describe a novel *c-myc* amplification in a unique liposarcoma in a patient with LFS.

**Case presentation:**

A female patient with LFS developed breast cancer twice at the age of thirty; both were invasive ductal carcinomas harboring *HER2* amplifications. Computed tomography revealed an anterior mediastinal mass, which was surgically resected. Histological analysis revealed three different lesions corresponding to myxoid liposarcoma-, pleomorphic liposarcoma-, and well-differentiated liposarcoma-like lesions. Fluorescence *in-situ* hybridization (FISH) analysis did not detect *MDM2* amplification, *Rb1* deletion, break apart signals of *EWS*, *FUS*, *DDIT3*, or *c-myc*, or *c-myc*-*IGH* fusion signals, but it did detect more *c-myc* signals. Further FISH analysis and comprehensive genomic profiling revealed *c-myc* amplification. We considered two differential diagnoses, dedifferentiated liposarcoma lacking *MDM2* amplification and myxoid pleomorphic liposarcoma (MPLPS), and determined that this case is most likely MPLPS. However, definite diagnosis could not be made because a clear-cut differentiation of the case from liposarcomas was not possible.

**Conclusions:**

A previous study demonstrated that *c-myc* amplification could not be detected in various liposarcomas, but the present unique liposarcoma showed *c-myc* amplification, so the *c-myc* amplification may indicate that the present liposarcoma is an LFS-related tumor. The present case further clarifies the pathological features of MPLPS and LFS-related liposarcomas by broadening their histopathological and genetic diversities.

## Background

Germline *TP53* mutations have been frequently reported in patients with Li–Fraumeni syndrome (LFS), resulting in a predisposition to various malignancies [1]. Mutations other than those of germline *TP53* can also cause LFS-associated malignancies, but their details have not been reported. The most common tumor arising in *TP 53* mutation carriers is breast cancer, followed by soft tissue sarcomas, brain tumors, osteosarcomas, and adrenocortical tumors [2–4]. Among sarcomas, osteosarcoma is the most common type, followed by sarcoma NOS (not otherwise specified), rhabdomyosarcoma, leiomyosarcoma, and liposarcoma [4, 5].

Liposarcomas (LPSs) are sarcomas of adipocytic differentiation. These tumors are pathologically classified into five subtypes: well-differentiated LPS (WLPS), dedifferentiated LPS (DLPS), myxoid LPS (MLPS), pleomorphic LPS (PLPS), and myxoid pleomorphic liposarcoma (MPLPS) [6]. Improved understanding of their underlying molecular pathologies, including *MDM2* amplification in WLPS and DLPS, fusion of *EWS*, *FUS,* and *DDIT3* in MLPS, and *Rb1* deletion in PLPS, has led to the development of subtype-tailored management and novel systemic therapies [6]. MPLPS is a rare but distinct tumor entity, which has been newly added to the latest World Health Organization (WHO) classification. Recently, its pathogenesis has been proposed to involve inactivation of *RB1* expression [7], and it lacks *MDM2* amplification and fusion of *EWS*, *FUS,* and *DDIT3* [8]. Furthermore, it is frequently associated with LFS [9]; however, our understanding of the molecular pathology of MPLPS is still limited, as there have been only a few case reports of MPLPSs.

Here, we describe a novel *c-myc* amplification in a unique MPLPS-like liposarcoma in a female patient with LFS using histological, immunohistochemical, and fluorescence *in-situ* hybridization **(**FISH) analysis and comprehensive genomic profiling (CGP).

## Case presentation

A female patient with LFS, diagnosed by DNA sequencing of a blood sample in which heterozygous pathogenic *TP53* mutations [1], c.818G > A (p.Arg273His), were identified, developed lymphocytic leukemia at the age of 11 and concurrent bilateral breast cancers (size, left: 11 mm × 6 mm, right: 25 mm × 13 mm) at the age of thirty. Lymphocytic leukemia remitted after chemotherapy, and both breast cancers were surgically resected and pathologically classified as invasive ductal carcinomas harboring immunohistochemically p53- and HER2-positive and *HER2* amplifications identified by FISH analysis, with no metastasis to axillar lymph nodes. No clinically significant mutation was identified by *BRCA1* and *BRCA2* analysis a the blood sample. The patient’s family history revealed that her mother developed left and right breast cancers, a brain tumor, and lymphoma before the age of 45, so that the patient clinically fulfilled the Clinical Criteria for Classic Li-Fraumeni syndrome [10].

**Fig. 1:**
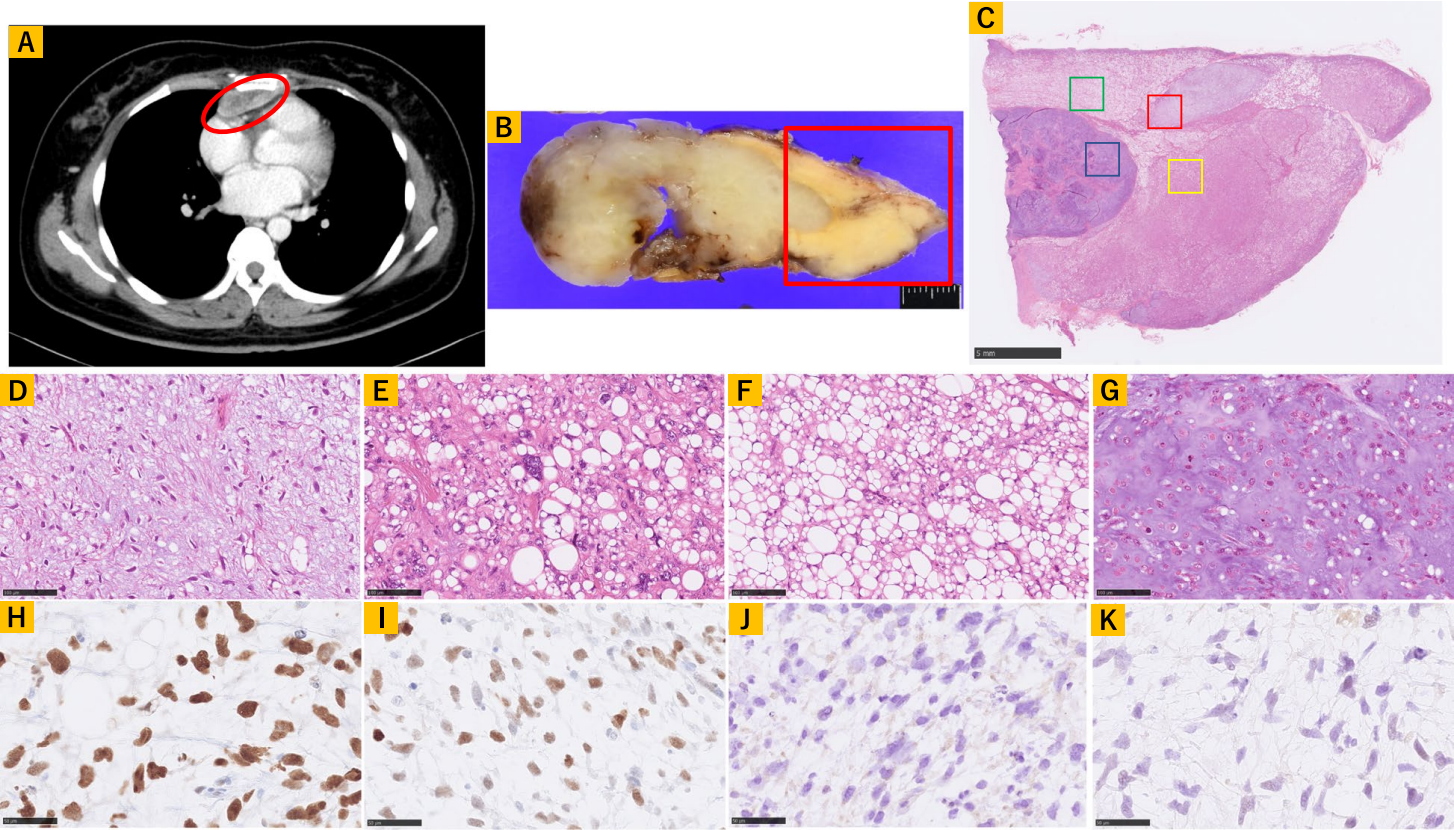
**A** Computed tomography reveals a mass (red circle), with a diameter measuring up to 5 cm, in the anterior mediastinum. **B** Gross image of the resected specimen showing a yellowish and whitish mass measuring 75 mm × 43 mm × 28 mm (scale bar = 10 mm). **C** Light micrograph of a hematoxylin–eosin-stained resected tissue specimen of the red square from Fig. 1B (scale bar = 5 mm). **D** High-power view of the selected area (red square) from Fig. 1C. Visible is a mixture of uniform round to oval-shaped non-lipogenic cells and small lipoblasts in a prominent myxoid stroma, similar to that of a myxoid liposarcoma (scale bar = 100 μm). **E** High-power view of the selected area (yellow square) from Fig. 1C, showing pleomorphic lipoblasts containing enlarged and hyperchromatic nuclei, similar to that of a pleomorphic liposarcoma (scale bar = 100 μm). **F** High-power view of the selected area (green square) from Fig. 1C, showing adipocytic proliferation with moderate nuclear atypia in adipocytes and stromal cells, which is difficult to use to make differential diagnosis between well-differentiated liposarcoma and pleomorphic liposarcoma (scale bar = 100 μm). **G** High-power view of the selected area (blue square) from Fig. 1C, showing nests of atypical chondroid cells that contain enlarged nuclei with a myxohyaline stroma (scale bar = 100 μm). Light micrographs of immunohistochemical staining for various proteins. **H** p53 and I) c-myc are expressed in nuclei of atypical cells (scale bar = 50 μm). **J** Rb1 and K) MDM2 are negative in the nuclei of atypical cells (scale bar = 50 μm)

About two weeks after breast lesions had been identified, computed tomography also revealed an anterior mediastinal mass (Fig. 1A), which was surgically resected. The resected specimen appeared yellow and white to gray on a relatively homogenous cut surface measuring 75 mm × 43 mm × 28 mm (Fig. 1B). Histological analysis revealed three different lesions in the yellow area (Fig. 1C), corresponding to a mixture of uniform round to oval-shaped non-lipogenic cells and small lipoblasts in the background of a prominent myxoid stroma, as observed in MLPS (Fig. 1D). We also identified pleomorphic lipoblasts harboring enlarged and hyperchromatic nuclei similar to those in PLPS (Fig. 1E). Furthermore, relatively mature-looking adipocytic proliferation showing moderate nuclear atypia in adipocytes and non-adipocytic stromal cells was detected, which is difficult to use to make differential diagnosis between WLPS of lipoma-like subtype and PLPS (Fig. 1F). Notably, the white lesion comprised nests of atypical chondroid cells harboring enlarged nuclei with a myxohyaline stroma (Fig. 1G).

Immunohistochemically, p53 (Fig. 1H) and c-myc (Fig. 1I) stained positive in the nuclei of these atypical cells, whereas both Rb1 (Fig. 1J) and MDM2 (Fig. 1K) were negative. The detailed protocol of immunohistochemistry is summarized in Table 1.

**Table 1:** Summary of the immunohistochemistry protocol

Antibody	Antigen retrieval treatment	Supplier	Dilution	Clone
p53	CC1, 64 min	Roche, Switzerland	Ready-to-use	DO7
c-myc	autoclave	Abcam, England	1;200	Y69
Rb1	autoclave, pH9	BD Pharmigen, US	1;1000	G3-245
MDM2	autoclave	Santa cruz, US	1;1000	SMP14

**Fig. 2:**
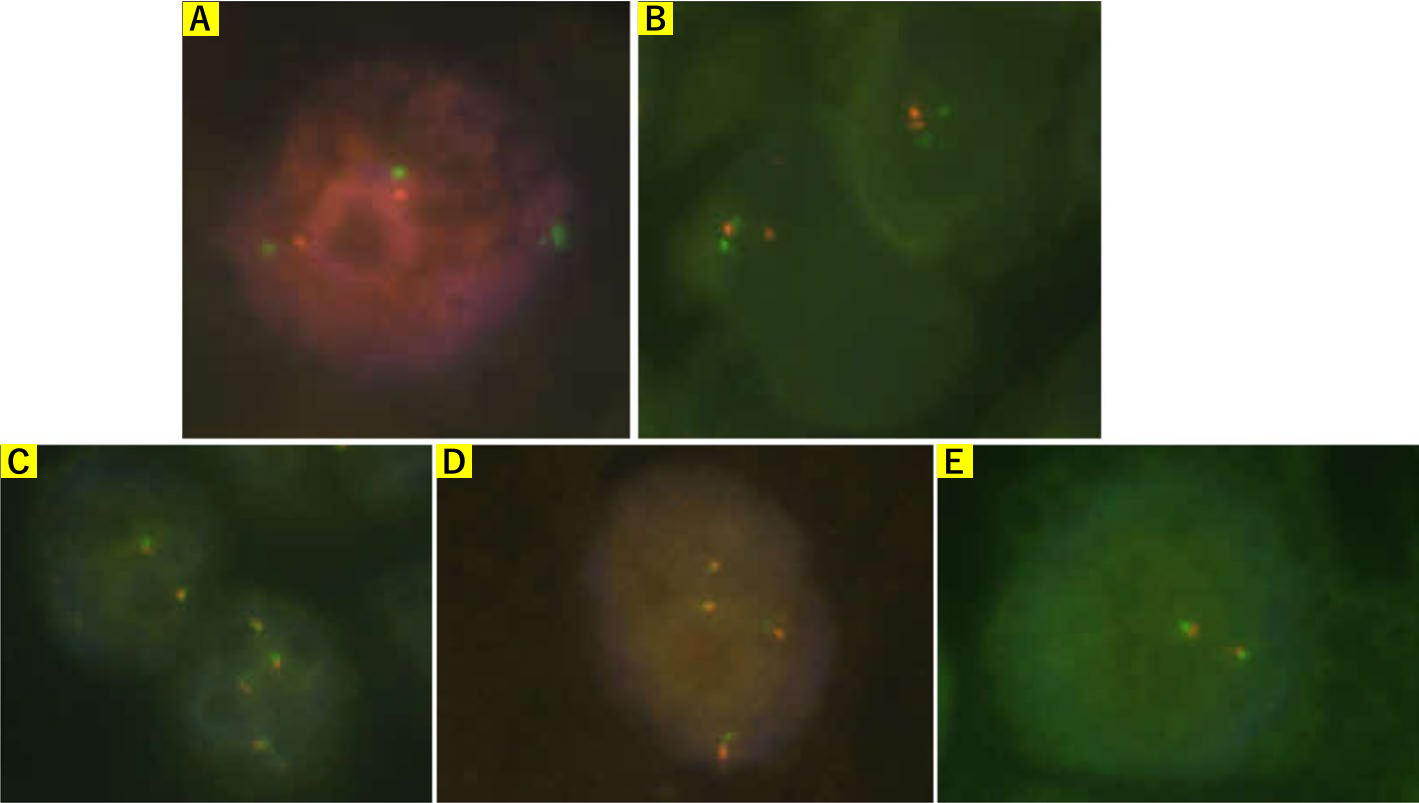
**A** FISH analysis using a Vysis LSI MDM2 Spectrum Orange Probe (Catalog Number: 1N1520, Abott, US) and a CEP12 DNA FISH probe (Catalog Number: 6J3722, Abott, US) did not show amplification signals of *MDM2* (orange: *MDM2*, green: *CEP12*). **B** FISH analysis using a ZytoLight SPEC RB1/13q12 Dual Color Probe (Product Number: Z-2165–200, ZytoVision, Germany) did not show loss of *Rb1* signals (orange: *Rb1*, green: *13q12*). **C** FISH analysis using Va ysis LSI EWSR1 Dual color Break Apart Probe (Catalog Number: 7J7101, Abott, US) did not show break-apart signals of *EWSR1* (orange: centromeric side, green: telomeric side). **D** FISH analysis using a Vysis LSI FUS Dual color, Break Apart Probe (Catalog Number: 7J6501, Abott, US) did not show break-apart signals of *FUS* (orange: centromeric side, green: telomeric side). **E** FISH analysis using a Vysis LSI DDIT3 Dual color Break Apart Probe (Product Number: 5J4805, Abbott, US) did not show break-apart signals of *DDIT3* (orange: centromeric side, green: telomeric side)

**Fig. 3:**
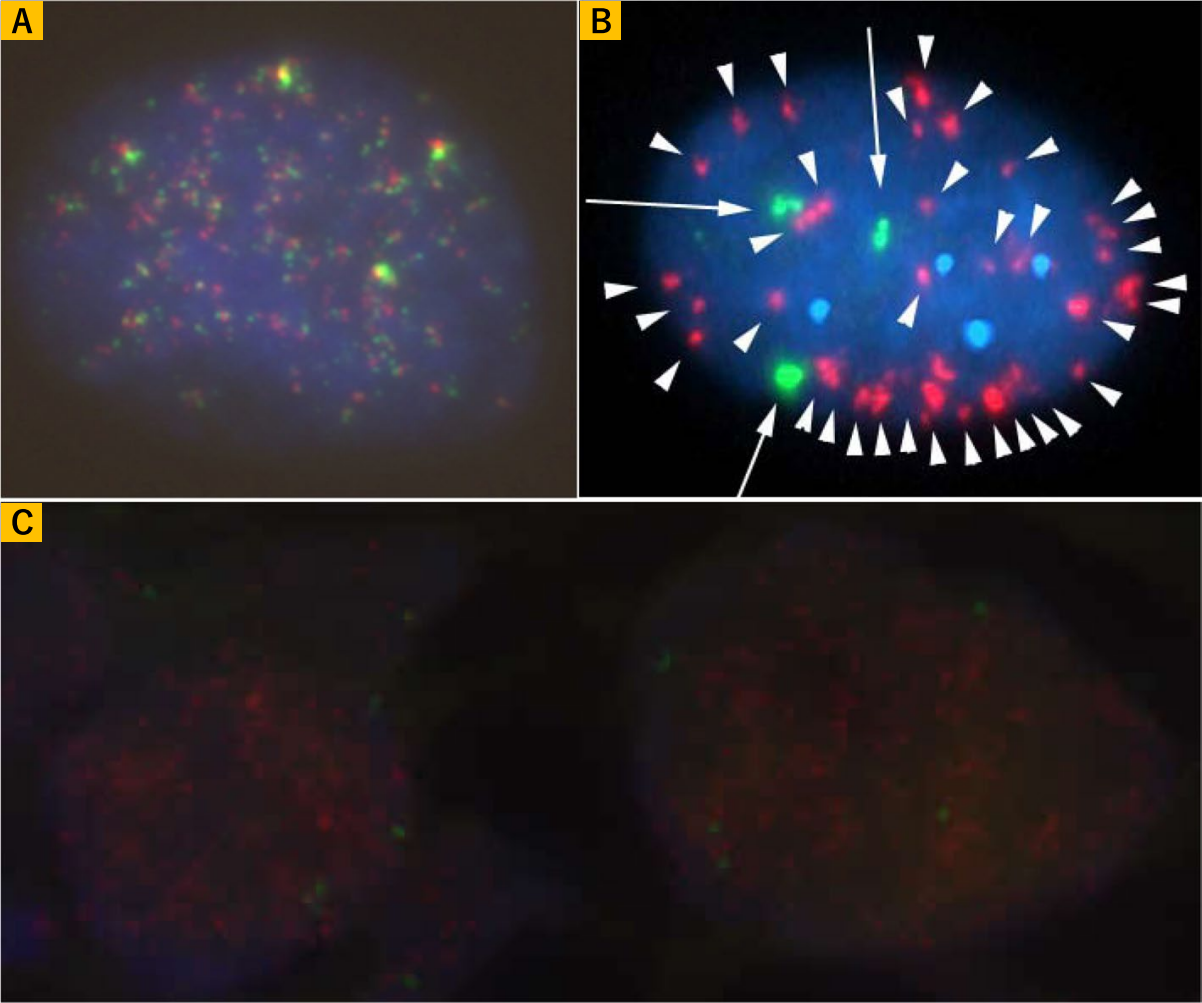
**A** FISH analysis using a Vysis LSI MYC Dual Color Break Apart Rearrangement Probe (Catalog Number: 5J9101, Abott, US) did not show break-apart signals of *c-myc*, but an increased number of *c-myc* signals (red: *5′ myc*, green: *3′ myc*). **B** FISH analysis using a Vysis LSI IGH/MYC CEP 8 Tri-color Dual Fusion Translocation Probe (Catalog Number: 5J7501, Abott, US) did not show fusion signals of *c-myc-IGH*, but an increased number of *c-myc* signals (red: *c-myc*, arrow head, green: *14q32*, arrow, blue: *8 cen*). **C** FISH analysis using a c-myc/CEN8p Dual Color FISH probe (Product Number: GC009, GSP Lab., Inc., Japan) showed amplification signals of *c-myc* (red: *c-myc*, green: CEN8p)

**Fig. 4:**
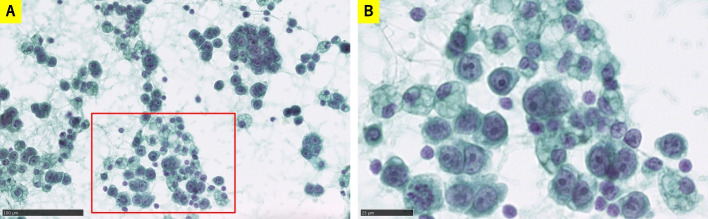
Cytology of pleural effusion. **A** Papanicolaou stain showing malignant cells with solitary or nested morphology (scale bar = 100 μm). **B** High-power view of the selected area (red square) from Fig. 3A, showing malignant cells with nuclear pleomorphism and multivacuolated cytoplasm (red arrow) like lipoblasts (scale bar = 25 μm)

FISH was performed using commercial probes to assess chromosomal abnormalities. The analysis did not detect *MDM2* amplification (Fig. 2A), *Rb1* deletion (Fig. 2B), break apart signals of *EWSR1* (Fig. 2C), *FUS* (Fig. 2D), *DDIT3* (Fig. 2E), or *c-myc* (Fig. 3A), or *c-myc*-*IGH* fusion signals (Fig. 3B), but it did detect more *c-myc* signals in 837 out of 996 tumor cells and 823 out of 1,000 tumor cells (Fig. 3A, B). Further FISH analysis using c-myc/CEN8p Dual Color FISH probe showed amplification of *c-myc* (Fig. 3C). Conclusively, our diagnosis of the mediastinal lesion in this patient was “Liposarcoma, most probably MPLPS showing cartilaginous differentiation.” Approximately ten months after the resection of the anterior mediastinal mass, magnetic resonance imaging again detected an anterior mediastinal mass supposed to be the recurrence of liposarcoma. CGP was performed using FOUNDATIONONE^®^CDx and formalin-fixed and paraffin-embedded sample of liposarcoma for the determination of the appropriate therapeutic strategy; however, no optimal therapeutic intervention could be implied from the results of CGP. The results of CGP were as follows: microsatellite status: stable; tumor mutational burden: low (3.78 Muts/Mb); *MYC* amplification observed (absolute copy number: 59); *Rb1* Splicing Variant c.1422-1G>T and *TP53* c.818G>A observed. As variants of unknown significance or benign variants, *BRAF* c.64G>A; *LTK* c638 639ins TGGCGG; *MST1R* c.679G>T; *NOTCH3* c.4039G>C; *TSC2* c.2409G>T; *SOX9* amplification (absolute copy number: 69); *MYC* rearrangement exon 2; *NOTCH3* rearrangement exon 1; and *SOX9* rearrangement exon 3 were detected. For recurrent liposarcoma, chemotherapy with gemcitabine and docetaxel was administered. However, pleural effusion appeared during this treatment, rendering it ineffective. Pleural effusion cytology detected malignant cells, suggesting liposarcoma (Fig. 4). Immunohistochemical analysis of sections from cell block samples of the pleural effusion revealed that malignant cells were vimentin-positive, S100-focal positive, and AE1/AE3-negative, which are consistent with liposarcoma. Further, chemotherapy with doxorubicin or eribulin was administered; however, the patient died approximately one year and four months after resectioning the anterior mediastinal mass.

## Discussion and conclusions

The nuclear accumulation of p53 in the present mediastinal tumor suggests that it arose in association with LFS [1]. In general, major liposarcomas, such as DLPS, MLPS, and PLPS, are clearly characterized by tumor-specific genetic alterations [6]. Therefore, amplification of *MDM2*, fusion of *EWS*, *FUS*, and *DDIT3*, and deletion of *Rb1* were examined in the present case [6, 11], but no such genetic abnormalities were demonstrated by FISH analyses. These results suggest that the three major subtypes are less probable from a genetic approach.

Histologically, the tumor consisted of heterogeneous components; MLPS-like, PLPS-like, and WLPS-like areas. Together with the abovementioned molecular findings, we considered two possible differential diagnoses: 1) DLPS lacking *MDM2* amplification and 2) MPLPS. As a DLPS, the PLPS-like area may correspond to homologous lipoblastic differentiation [12], although such differentiation is relatively rare. In addition, this tumor lacked a definite well-differentiated component, which is quite exceptional for DLPS. Notably, most DLPSs harbor the amplification of *MDM2* [6, 11], but negative cases have been previously reported in the settings of LFS with abnormal p53 status [13]. Collectively, we considered that DLPS is unlikely, but cannot be completely excluded. Conversely, MPLPS, newly classified in 2020 as a soft tissue and bone tumor by the WHO, is common in children and young adults under 30 years of age [14] and associated with LFS [9]. Moreover, MPLPS has a predilection for the mediastinum [14], showing a mixture of PLPS-like and MLPS-like components, as in our case. Recently, its pathogenesis has been proposed to involve inactivation of *RB1* expression [7]. In the present case, Rb1 was negative immunohistochemically and *Rb1* deletion was not detected; thus, the *Rb1* splicing variant c.1422-1G>T detected by CGP was supposed to be associated with Rb1 inactivation. The lack of *MDM2* amplification and fusion of *EWS*, *FUS,* and *DDIT3* was consistent with MPLPS [8]. To the best of our knowledge, cartilaginous differentiation has not been reported in MPLPS cases, but has been reported in various sarcomas, including lipomatous tumors, such as WLPS, DLPS, and MLPS [15, 16]. Therefore, cartilaginous differentiation is possibly a non-specific finding for MPLPS, which is the same as for other liposarcomas. We considered that this unique liposarcoma falls into the category of MPLPS, which shows peculiar cartilaginous differentiation, but definite diagnosis as MPLPS was not possible because DLPS could not be excluded.

We detected a *c-myc* amplification in the present tumor, in addition to germline *TP53* mutations. *C-myc* is frequently dysregulated in various neoplasms, and its amplification has also been reported in the tumor from patients with LFS [2]; its overexpression drives metabolic changes and increases cellular proliferation [17, 18]. Previous studies suggested that *TP53* mutation was associated with various oncogene amplifications [19, 20]. In addition, *c-myc* amplification could not be detected in various liposarcomas in a past study [21], but the present unique liposarcoma showed *c-myc* amplification, so the *c-myc* amplification may indicate that the present liposarcoma is an LFS-related tumor. Furthermore, in various sarcomas, including liposarcomas, c-myc overexpression was reported to be correlated with poor prognosis [21–23], and the patient died approximately one year and four months later after resection; therefore, c-myc overexpression observed in the present tumor may also be associated with poor prognosis.

In summary, we identified a novel *c-myc* amplification in a unique liposarcoma in an LFS patient. The present case further clarifies the pathological features of MPLPS and LFS-related liposarcomas by broadening their histopathological and genetic diversities.

## Data Availability

Not applicable.

## Abbreviations

FISH: Fluorescence *in-situ* hybridization
LFS: Li–Fraumeni syndrome
LPSs: Liposarcomas
WLPS: Well-differentiated LPS
DLPS: Dedifferentiated LPS
MLPS: Myxoid LPS
PLPS: Pleomorphic LPS
MPLPS: Myxoid pleomorphic liposarcoma
WHO: World Health Organization
CGP: Comprehensive genomic profiling

## References

[1] Malkin D, Li FP, Strong LC, Fraumeni JF, Nelson CE, Kim DH (1990). Germ line p53 mutations in a familial syndrome of breast cancer, sarcomas, and other neoplasms. Science.

[2] Rieber J, Remke M, Hartmann C, Korshunov A, Burkhardt B, Sturm D (2009). Novel oncogene amplifications in tumors from a family with Li–Fraumeni syndrome. Genes Chromosomes Cancer.

[3] Masciari S, Dillon DA, Rath M, Robson M, Weitzel JN, Balmana J (2012). Breast cancer phenotype in women with TP53 germline mutations: a Li–Fraumeni syndrome consortium effort. Breast Cancer Res Treat.

[4] Bouaoun L, Sonkin D, Ardin M, Hollstein M, Byrnes G, Zavadil J (2016). TP53 variations in human cancers: new lessons from the IARC TP53 database and genomics data. Hum Mutat.

[5] Ognjanovic S, Olivier M, Bergemann TL, Hainaut P (2012). Sarcomas in TP53 germline mutation carriers: a review of the IARC TP53 database. Cancer.

[6] Lee ATJ, Thway K, Huang PH, Jones RL (2018). Clinical and molecular spectrum of liposarcoma. J Clin Oncol.

[7] Hofvander J, Jo VY, Ghanei I, Gisselsson D, Mårtensson E, Mertens F (2016). Comprehensive genetic analysis of a paediatric pleomorphic myxoid liposarcoma reveals near-haploidization and loss of the RB1 gene. Histopathology.

[8] Creytens D, van Gorp J, Ferdinande L, Van Roy N, Libbrecht L (2014). Array-based comparative genomic hybridization analysis of a pleomorphic myxoid liposarcoma. J Clin Pathol.

[9] Sinclair TJ, Thorson CM, Alvarez E, Tan S, Spunt SL, Chao SD (2017). Pleomorphic myxoid liposarcoma in an adolescent with Li–Fraumeni syndrome. Pediatr Surg Int.

[10] Gonzalez KD, Noltner KA, Buzin CH, Gu D, Wen-Fong CY, Nguyen VQ (2009). Beyond Li Fraumeni Syndrome: clinical characteristics of families with p53 germline mutations. J Clin Oncol.

[11] Binh MB, Sastre-Garau X, Guillou L, de Pinieux G, Terrier P, Lagacé R (2005). MDM2 and CDK4 immunostainings are useful adjuncts in diagnosing well-differentiated and dedifferentiated liposarcoma subtypes: a comparative analysis of 559 soft tissue neoplasms with genetic data. Am J Surg Pathol.

[12] Boland JM, Weiss SW, Oliveira AM, Erickson-Johnson ML, Folpe AL (2010). Liposarcomas with mixed well-differentiated and pleomorphic features: a clinicopathologic study of 12 cases. Am J Surg Pathol.

[13] Debelenko LV, Perez-Atayde AR, Dubois SG, Grier HE, Pai SY, Shamberger RC (2010). p53+/mdm2- atypical lipomatous tumor/well-differentiated liposarcoma in young children: an early expression of Li–Fraumeni syndrome. Pediatr Dev Pathol.

[14] Alaggio R, Coffin CM, Weiss SW, Bridge JA, Issakov J, Oliveira AM (2009). Liposarcomas in young patients: a study of 82 cases occurring in patients younger than 22 years of age. Am J Surg Pathol.

[15] Siebert JD, Williams RP, Pulitzer DR (1996). Myxoid liposarcoma with cartilaginous differentiation. Mod Pathol.

[16] Al-Rikabi AC, El-Sharkawy MS, Al-Seif A (2013). Primary well differentiated breast liposarcoma with divergent cartilagenous differentiation: a case report. Oman Med J.

[17] Miller DM, Thomas SD, Islam A, Muench D, Sedoris K (2012). c-Myc and cancer metabolism. Clin Cancer Res.

[18] Prochownik EV (2004). c-Myc as a therapeutic target in cancer. Expert Rev Anticancer Ther.

[19] Sugawara W, Arai Y, Kasai F, Fujiwara Y, Haruta M, Hosaka R (2011). Association of germline or somatic TP53 missense mutation with oncogene amplification in tumors developed in patients with Li–Fraumeni or Li–Fraumeni-like syndrome. Genes Chromosomes Cancer.

[20] Bull SB, Ozcelik H, Pinnaduwage D, Blackstein ME, Sutherland DA, Pritchard KI (2004). The combination of p53 mutation and neu/erbB-2 amplification is associated with poor survival in node-negative breast cancer. J Clin Oncol.

[21] Tran D, Verma K, Ward K, Diaz D, Kataria E, Torabi A (2015). Functional genomics analysis reveals a MYC signature associated with a poor clinical prognosis in liposarcomas. Am J Pathol.

[22] Tsiatis AC, Herceg ME, Keedy VL, Halpern JL, Holt GE, Schwartz HS (2009). Prognostic significance of c-Myc expression in soft tissue leiomyosarcoma. Mod Pathol.

[23] Scionti I, Michelacci F, Pasello M, Hattinger CM, Alberghini M, Manara MC (2008). Clinical impact of the methotrexate resistance-associated genes C-MYC and dihydrofolate reductase (DHFR) in high-grade osteosarcoma. Ann Oncol.

